# New Textile Sensors for In Situ Structural Health Monitoring of Textile Reinforced Thermoplastic Composites Based on the Conductive Poly(3,4-ethylenedioxythiophene)-poly(styrenesulfonate) Polymer Complex

**DOI:** 10.3390/s17102297

**Published:** 2017-10-10

**Authors:** Ivona Jerkovic, Vladan Koncar, Ana Marija Grancaric

**Affiliations:** 1 Department of Textile Chemistry and Ecology, University of Zagreb Faculty of Textile Technology, 10000 Zagreb, Croatia; amgranca@ttf.hr; 2 Ecole Nationale Supérieure des Arts et Industries Textiles, GEMTEX Laboratory, 59056 Roubaix, France; vladan.koncar@ensait.fr

**Keywords:** textile sensors, conducting polymers, textile-reinforced composites, electromechanical properties, gauge factor

## Abstract

Many metallic structural and non-structural parts used in the transportation industry can be replaced by textile-reinforced composites. Composites made from a polymeric matrix and fibrous reinforcement have been increasingly studied during the last decade. On the other hand, the fast development of smart textile structures seems to be a very promising solution for in situ structural health monitoring of composite parts. In order to optimize composites’ quality and their lifetime all the production steps have to be monitored in real time. Textile sensors embedded in the composite reinforcement and having the same mechanical properties as the yarns used to make the reinforcement exhibit actuating and sensing capabilities. This paper presents a new generation of textile fibrous sensors based on the conductive polymer complex poly(3,4-ethylenedioxythiophene)-poly(styrenesulfonate) developed by an original roll to roll coating method. Conductive coating for yarn treatment was defined according to the preliminary study of percolation threshold of this polymer complex. The percolation threshold determination was based on conductive dry films’ electrical properties analysis, in order to develop highly sensitive sensors. A novel laboratory equipment was designed and produced for yarn coating to ensure effective and equally distributed coating of electroconductive polymer without distortion of textile properties. The electromechanical properties of the textile fibrous sensors confirmed their suitability for in situ structural damages detection of textile reinforced thermoplastic composites in real time.

## 1. Introduction

Many metallic structural and non-structural parts used in the transportation industry could be replaced by textile-reinforced composites where each production step has to be monitored to obtain high tech products. These composites have to be produced to meet technical performance specifications, weight reduction, recyclability and market requirements [1,2,3,4,5,6,7,8,9,10]. Composites made from a polymeric matrix and a fibrous reinforcement have been increasingly studied during the last decade due to their remarkable features such as corrosion, chemical and impact resistance, dimensional stability, design flexibility, suitable electromagnetic properties, temperature tolerance, etc. [11,12]. In composite applications, the low material density is of environmental interest because fuel consumption and CO_2_ emissions are directly related to vehicle weight [8,13].

The role of the interface needs to be also taken into account in composite structure-property relationship requirements [8,10,11]. The interface is defined as the three-dimensional region surrounding reinforcement yarns, in contact with the matrix, with its own characteristics, corresponding neither to yarn properties nor to matrix ones [10]. A better interfacial bond between fibre and matrix gives better properties to the composites, such as the interlaminar shear strength and delamination [14].

Textile-reinforced thermoplastic composites maintain good tolerances, material strengths and show distinct advantages over thermoset composites like lower density, unlimited storage, semi-products delivered ready for use, thermoformability, a faster processing cycle, no solvent emissions during the processing stage, etc. Furthermore, concerning their high fracture toughness, recycling possibility, various forms, high damage tolerance and resistance to medias and corrosion, they appear to be more promising for industrial applications. These composites are consumed in large volume, especially in the transportation industry for automotive, railway and truck component part production [13,15,16,17,18,19,20,21,22].

Composites are more complex than their metallic counter parts due to non-homogeneous structures. Their durability and safety issues are more important [23]. For metals, a predominant single crack is the most common failure mechanism. In composites, there are four basic failure mechanisms: (i) fibre breakage; (ii) delamination; (iii) cracking and (iv) interfacial debonding [6,15].

The usage of smart textile structures in order to realize textile fibrous sensors compatible with composite technology is a very promising solution for in situ structural health monitoring of composite parts. Such smart materials could be made by coating or treating textile filaments, yarns, or fabrics with nanoparticles or conductive and semi-conductive polymers, giving them specific performance characteristics [24].

Smart textiles play a significant role in the European textile sector and have helped the textile industry in its transformation into a competitive knowledge-driven industry. These kinds of textiles combine knowledge from many disciplines with the specific textile requirements [25].

Textile sensors are a large class of smart textiles in general, typically found in medical applications [1,2,3,4,26,27,28,29]. These sensors perform a dual function inside a composite. After integration in the reinforcement, they act as a part of structural material and have actuating and sensing capabilities. Their working principle originally relied on the traditional metal-based strain gauges [30,31]. In general, strain gauges for textiles are based on electrically resistive materials or structures whose electrical resistance changes reversibly according to an applied stress. The term “piezo-resistive sensor” is commonly used and its development is the main objective of this study [1,2,32,33,34,35,36]. Recently, interest has focused on the possibility to develop these sensors from Intrinsically Conductive Polymers (ICPs) also called “synthetic metals”. 

In the late 1970s, Heeger, MacDiarmid and Shirakawa discovered how to get polymers that conduct electricity. These polymers resulted in a paradigmatic change in scientific thinking and opened new frontiers in chemistry, physics and materials science. The first material used as an intrinsically conductive polymer was polyethyne (other name polyacetylene (PAc)), after a doping with iodine. The announcement of this discovery quickly reverberated around the scientific community, and the intensity of research seeking other conductive polymers magnified dramatically [2].

ICPs are composed of polymer chains containing alternating single and double bonds called conjugated double bonds. Electrons are able to move from one end of the polymer to the other through the extended p-orbital system [1,2,32,33,34,35,36]. They can be applied to the surface of various substrates by using different techniques, such as dip-coating, solution casting, inkjet printing, 3D printing, etc. [2,22].

Unlike metals, their conductivity increases with temperature similarly to amorphous semiconductors. Variable electrical conductivity, electroactive properties and the ability to produce these polymers at low cost have led to investigations of their potential applications such as electromagnetic shielding, corrosion protection, radar absorption, sensing, actuating, thin film transistors, organic light emitting diodes, supercapacitors, organic solar cells and electrochromic displays [34,37,38].

Polypyrrole (PPy), polythiophene (PTh), polyaniline (PANI) and poly(3,4-ethylenedioxythiophene) (PEDOT) offer the best compromise between stability and processability and a broad range of electrical conductivity from 10^−10^ to 10^+6^ Scm^−1^ [1,2,32,33,34,35,36].

Amongst the wide range of ICPs PEDOT is one of the most promising conducting polymers due to its interesting properties such as excellent transparency in the visible range, high conductivity (>300 Scm^−1^) and good thermal stability [39,40]. Like other ICPs, PEDOT has a stiff conjugated aromatic backbone structure, which makes it insoluble in most organic and inorganic solvents.

Polystyrene sulfonic acid (PSS), a water-soluble polyanion, is used during the polymerization of PEDOT as a charge balancing dopant. Electrostatic interactions between the charged sulfonate groups on the PSS backbone and the PEDOT backbone occur [39,40,41,42,43]. PSS allows the dispersion of the PEDOT in water generating a complex where the oligomeric PEDOT segments are attached to the long chains of the PSS [44]. The PEDOT:PSS gel particles have excellent film-forming properties and are easily processable into thin coatings on a variety of substrates. This coating processability has facilitated the widespread availability of PEDOT:PSS as a commercially useful polymer complex for the production of highly transparent conductive polymer coatings with high mechanical flexibility, excellent chemical (environmental) and thermal stability, ease of synthesis [45,46,47]. The ionic species PEDOT+ and PSS- could not be separated by standard capillary electrophoresis methods [39,43,47,48,49]. However, PSS itself is a non-conducting polymer, which limits the conductivity of the polymer complex PEDOT:PSS to the 1–10 Scm^−1^ range [39,47,48,50]. 

Today ICPs based on PEDOT are commercially available in large quantities [47]. PEDOT:PSS has been widely used as an electrode material in organic thin film transistors or as a hole transport layer in organic light emitting diodes. This polymer complex can coat hard surfaces of microelectronics as well as fibres and fabrics and other stretchable substrates [51]. 

Due to its interesting electromechanical properties and a possibility to be used as a coating material the PEDOT:PSS polymer complex has been selected in this work for the development of a new generation of textile fibrous sensors adapted for in situ Structural Health Monitoring (SHM) of textile-reinforced thermoplastic composites.

Percolation threshold determination of this polymer complex was based on conductive dry films’ electrical properties analysis in order to develop highly sensitive sensors to detect small deformations occurring within composite structure and to guarantee their optimal functioning.

A novel piece of laboratory equipment based on a conceptual design study has also been produced to ensure effective and equally distibuted coating of electroconductive polymer without distortion of textile properties. 

The final objective of this paper focuses on the realization of predictive maintenance concept. The electromechanical properties of textile fibrous sensors were observed to validate their suitability for in situ structural damages detection of textile-reinforced thermoplastic composites in real time. 

## 2. Experimental

### 2.1. Materials and Methods 

The fibrous sensors developed in this study are based on functionalization of commingled yarns that have been used for the manufacturing of reinforcements for composite structures. The functionalization made of reinforcement yarns as strain gauge sensors is locally on the areas that have been coated. This methodology is important because it enables the reinforcement yarns deformations measurements on the real “reinforcement” yarns and gives accurate results on their deformations. Also, the functionalization procedure should not modify reinforcement yarns mechanical properties. 

E-glass/polypropylene (GF/PP) commingled yarn, E-glass/polyamide66 (GF/PA66) commingled yarn and E-glass (GF) yarn produced by PD Fiberglass group (Glasseiden GmbH, Oschatz, Germany) were used for textile sensors development presented in Table 1. The list of chemicals and other materials needed for their production are shown in Table 2 and Table 3.

The coating thickness and uniform distribution are very important parameters having diverse effects on the properties of textile sensors developed and consequently on end-user applications. Yarn treated only with aqueous dispersion of polymer complex PEDOT:PSS is too brittle during the tensile test according to previous investigations [37].

Therefore, an aqueous dispersion of PEDOT:PSS polymer complex, CLEVIOS P FORM. CPP105D (A) or CLEVIOS F ET (B), and synthetic latex, Latex Appretan 96100 (C), were combined (Table 4).

Aqueous dispersion of polymer complex PEDOT:PSS consists of sub-micrometre-sized gel particles which upon drying can form a continuous film which is both conductive and transparent [52,53].

According to the results of preliminary studies, A/B mixture was stirred at 50 °C until 40% solvent evaporation while B/C mixture at 50 °C until 25% solvent evaporation to increase its viscosity and conductivity [54,55,56,57]. The speed of dispersion mixing was 550 rpm the first 30 min and after that 1100 rpm until needed solvent evaporation (ca. 4 h).

**Figure 1:**
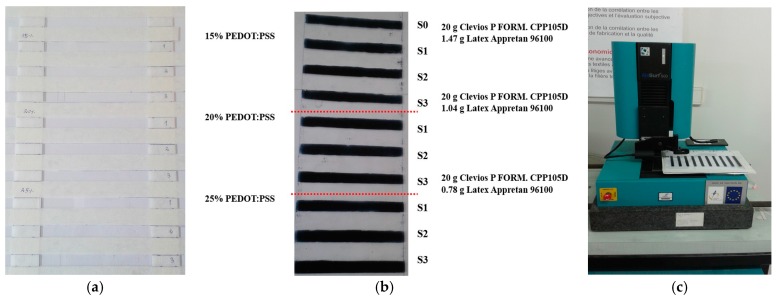
Conductive polymer films preparation for percolation threshold study: (**a**) frame preparation; (**b**) dry PEDOT:PSS films; (**c**) optical profilometer.

The polymer films for electrical resistance testing were prepared by delivering 500 μL of dispersion(s) by micropipette corresponding to the various content of PEDOT:PSS polymer complex (Table 5) to frames placed on a plexiglass surface (Figure 1). These frames were based on cellulose acetate tracks with dimensions 100 mm × 10 mm (track length × track width, *L* × *l*). Three polymer films were realized for each formulation.

After 48 h of solvent evaporation the thicknesses of the dry films were determined by an optical profilometer (Altisurf 500, Altimet SAS, Thonon-les-Bains, France). 

Dry films placed on the plexiglass surface were positioned under the measuring head. A scan of each dry film was performed to record its surface roughness, from which the thickness of each dry film was deduced. Thickness of each dry film [3,58,59,60,61] is an average of ten profiles measured along the track. 

Final thickness for each conductive formulation was calculated as an average of three films per previously mentioned formulation.

#### 2.1.1. Electrical Resistance and Resistivity of Conductive Dry Films

**Figure 2:**
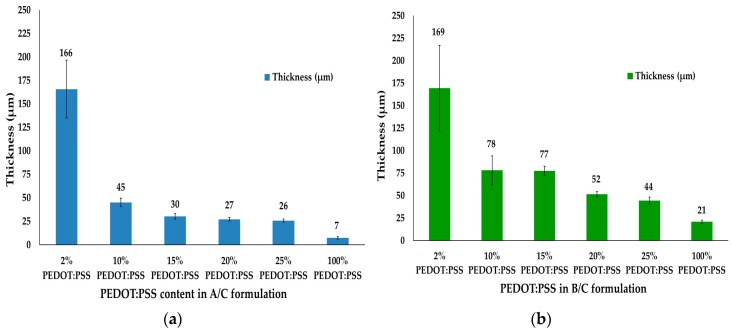
Conductive dry films (tracks) thicknesses ranging for different content of polymer complex PEDOT:PSS in aqueous conductive dispersion: (**a**) A/C formulation; (**b**) B/C formulation.

Conductive dry films were realized with different thicknesses ranging from 7 to 166 μm for A/C formulations and from 21 to 169 μm for B/C formulations depending on the content of PEDOT:PSS polymer complex (Figure 2).

The thickness of these films (tracks) could not be presumed uniformly planar, the standard deviation is taken into account based on ten profiles measured (observed) along the track for three films per formulation, previously mentioned [43].

**Figure 3:**
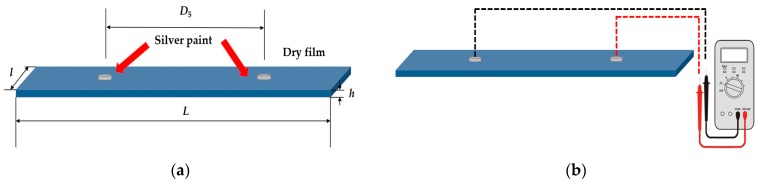
Conductive dry films preparation (**a**) and electrical resistance measurements by a standard Ohmmeter at distance of 5 cm between silver points (**b**).

Silver drops (RS Components) were placed at 5 cm distance (*D*_5_) at each dry film (Figure 3a). The electrical resistances of conductive dry films were measured by a standard Ohmmeter after 6, 8, 12, 65, 70 and 75 days (Figure 3b) to analyze their electrical resistances and related electrical resistivity changes versus time. 

Silver paint is used for various applications including to paint-on an electrical screen, or to make electrical connections to non-solderable surfaces. Its application is simple with a brush and is touch-dry in 10 min and usable in 30 min.

**Figure 4:**
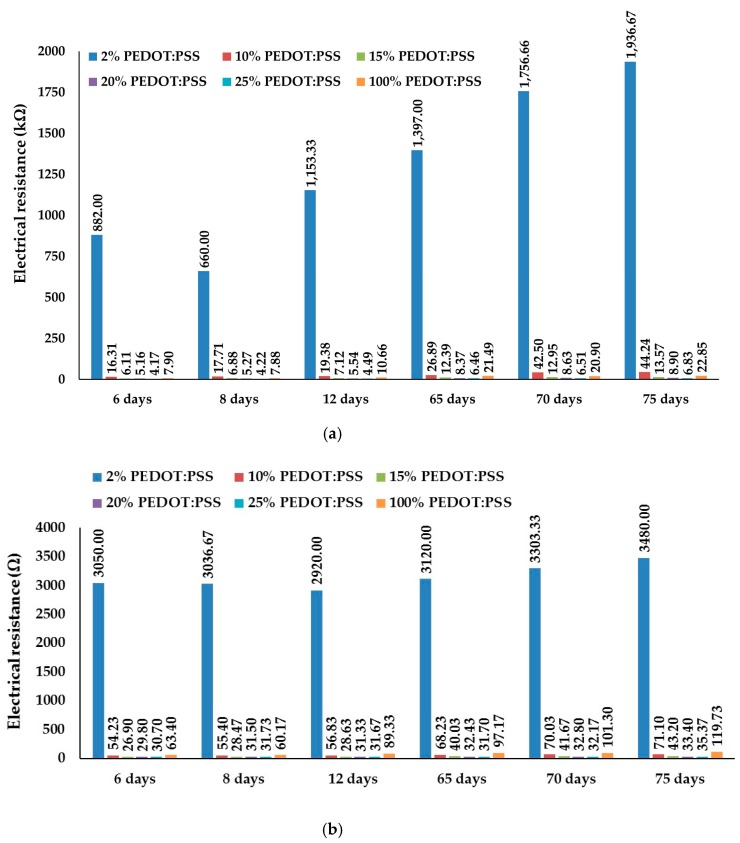
Electrical resistance of conductive dry films: (**a**) A/C dry film; (**b**) B/C dry film.

The evolutions of the electrical resistances after 6, 8, 12, 65, 70 and 75 days of conductive dry films monitoring at distance of 5 cm between silver points are presented in Figure 4.

Following properties related to electrical resistance have been observed:

(i) The electrical resistances of 2–25% PEDOT:PSS conductive dry films increased when the track thicknesses decreased depending on the PEDOT:PSS content in the prepared A/C or B/C formulations. The electrical resistances also increased with the decrease in thickness for the neat PEDOT:PSS film compositions.
(ii) The electrical resistances decrease with the increase in the PEDOT:PSS content in dispersions.
(iii) Electrical resistance of dry films increased with time for 2–25% PEDOT:PSS formulations. There is an insignificant electrical resistance decrease afterwards:
  - 8 days compared to 6 days for 2% PEDOT:PSS A/C dry film
  - 8 and 12 days compared to 6 days for 2% B/C dry film
  - 20 days compared to 12 days for 20% PEDOT:PSS A/C dry film
  - 12 and 65 days compared to 8 days for 25% PEDOT:PSS B/C dry film
(iv) Electrical resistance of conductive dry films also increased with time for 100% PEDOT:PSS dry films with insignificant electrical resistance decrease afterwards:
  - 8 days compared to 6 days for 100% PEDOT:PSS A dry film
  - 70 days compared to 65 days for 100% PEDOT:PSS A dry film
  - 8 days compared to 6 days for 100% PEDOT:PSS B dry film

Higher changes in electrical resistance of mostly all dry films can be observed after 65 days. 100% PEDOT:PSS B-dry films are more stable during observed period than 100% PEDOT:PSS A-dry films. 

Finally, the electrical resistivity and related electrical conductivity calculations of conductive dry films gave more precise data analysis. The electrical resistivity of dry film, *ρ* (Ω·m), is calculated from, *R*, the electrical resistance (Ω), *D*, the distance between silver points (m), *l*, the width of the track (m) and, *h*, the thickness of the track (m) (Equation (1)):

(1) $$\rho =\frac{R\cdot l\cdot h}{D}$$

The electrical conductivity, *σ* (Sm^−1^), is the reciprocal value of the electrical resistivity, *ρ*, (Ω·m) (Equation (2)):

(2) $$\sigma =\frac{1}{\rho }$$

#### 2.1.2. Percolation Threshold

The electrical resistivity of conductive dry films is an important parameter for the percolation threshold determination of A/C and B/C formulations in order to define the appropriate PEDOT:PSS content for textile sensor development. The critical amount of the conductive filler to form continuous conductive paths or networks and cause a dramatic resistivity decrease is known as the percolation threshold [62,63].

**Figure 5:**
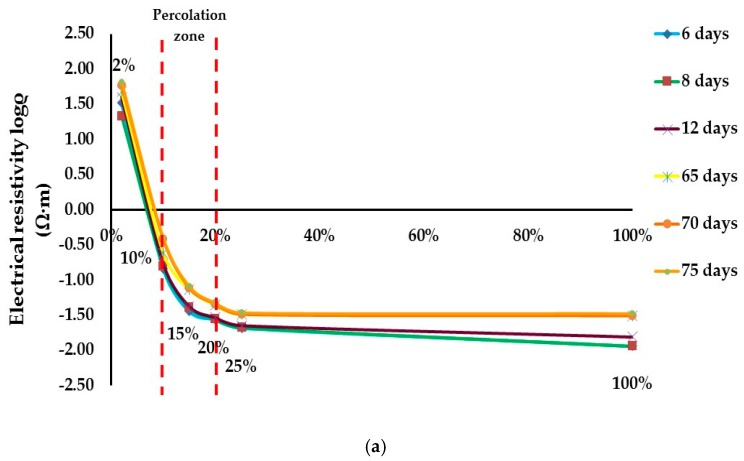

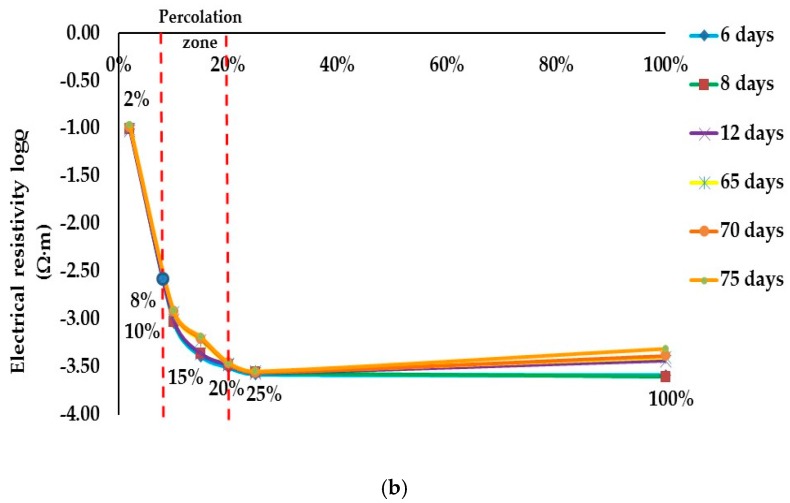
Percolation threshold study of PEDOT:PSS content (%) in a period of 75 days: (**a**) A/C formulation; (**b**) B/C formulation.

Electrical resistivity changes versus PEDOT:PSS content in A/C or B/C formulations (conductive dispersions) after 6, 8, 12, 65, 70 and 75 days of dry films monitoring at distance of 5 cm between silver points are presented in Figure 5.

The percolation threshold has been determined in the left part of the slope for both aqueous conductive dispersions. In general, there are no mathematical methods to determine the percolation threshold [1]. In order to determine it properly, the percolation zone has been identified.

For PEDOT:PSS A/C formulation, it ranges from 10% to 20%. This zone corresponds to the sharp modification of the electrical resistivity, then the average value—15%—is taken as a percolation threshold.

For PEDOT:PSS B/C formulation, the percolation zone is also going from the concentration of less than 10% up to 20%. In this case we have decided to define the percolation threshold at the beginning of the percolation zone that is 8% in order to have more sensitive sensor for small deformation measurements, and to verify its behaviour with rather low concentration of PEDOT:PSS.

**Figure 6:**
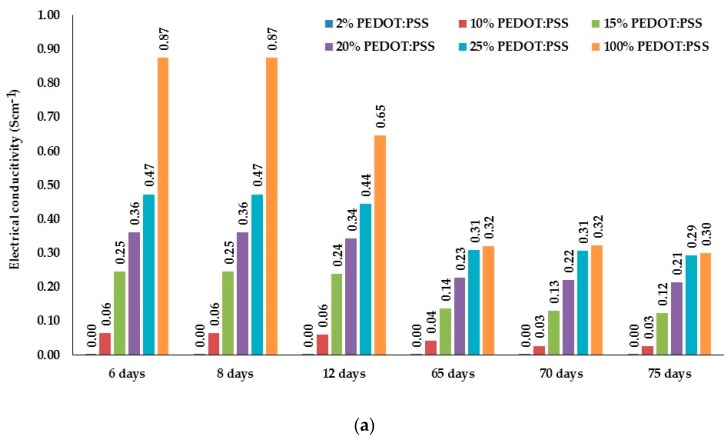

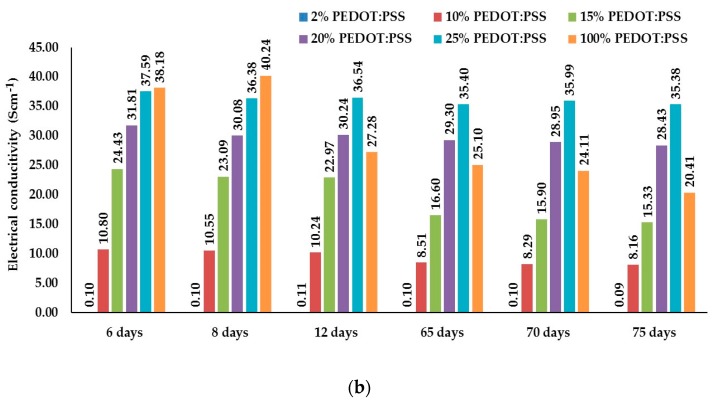
Electrical conductivity (S cm^−1^) of A/C and B/C dry films in a period of 75 days: (**a**) A/C dry films; (**b**) B/C dry films.

The ratio for the first conductive formulation corresponds to a PEDOT:PSS/C ratio of 15:85 studied by monitoring of mixture A/C, 20 g of chemical A and 1.47 g of chemical C, while for the second formulation the PEDOT:PSS/C ratio corresponds to 08:92 by monitoring of mixture B/C, 20 g of chemical B and 7.13 g of chemical C, during its stirring under strict conditions previously mentioned. Electrical conductivity of dry films is presented in Scm^−1^ (Figure 6). Electrical conductivity of both formulations after preparation started to decrease progressively in observed period of 75 days. A-dry films are less conductive compared to B-dry films. Non-conductive aqueous dispersion, chemical C, gives additional stability of developed conductive dry films after 12 days. 25% PEDOT:PSS B/C dry film is even more stable and electrically conductive compared to 100% PEDOT:PSS B-dry film.

**Figure 7:**
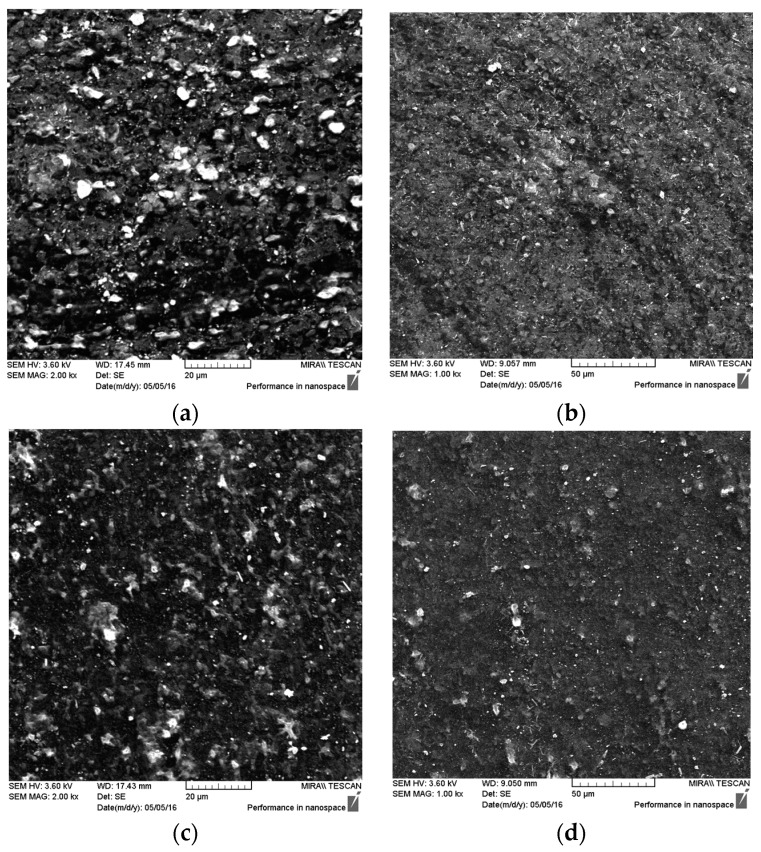
Scanning electron micrographs of conductive dry films: (**a,b**) 15% PEDOT:PSS A/C dry film and (**c,d**) 8% PEDOT:PSS B/C dry film.

Scanning electron micrographs of conductive dry films, 15% PEDOT:PSS A/C dry film (thickness 94.90 μm) and 8% PEDOT:PSS B/C dry film (thickness 135.67 μm) are presented in Figure 7. Both conductive dry films have granular morphology. The second dry film (Figure 7c,d) shows a more homogeneous surface, justifying its better electrical conductivity. Hence, higher electrical conductivity may be attributed to uniformity of the coating.

### 2.2. Textile Sensors Production

**Figure 8:**
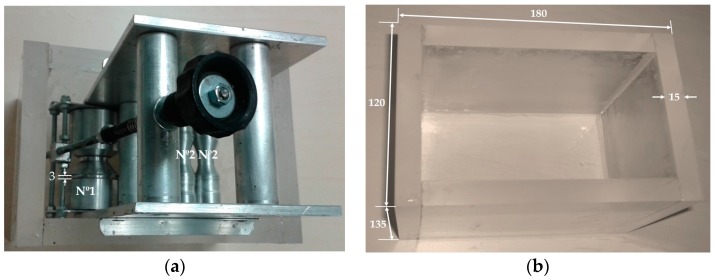
Developed system for roll to roll coating method: (**a**) aluminum roll to roll laboratory device with plexiglass chamber; (**b**) plexiglass chamber.

**Figure A1:**
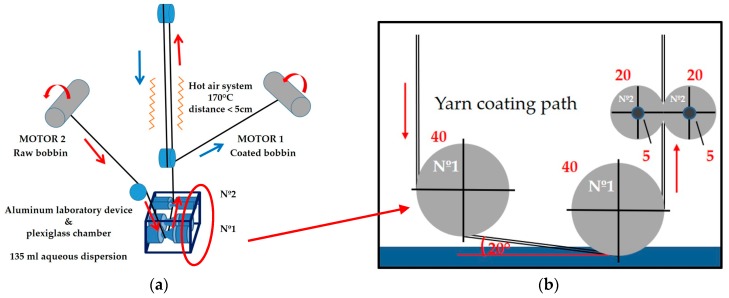
Roll to roll coating—scheme: (**a**) process; (**b**) aluminum laboratory device—lateral side.

According to the design concept study developed in our studies [64] and presented in Appendix A (Figure A1) an aluminum roll to roll laboratory device and plexiglass chamber (Figure 8) were realized and used for fibrous sensor production, guaranteeing good quality and optimal coating repeatability.

After partially yarn coating at the center of the sample, the yarn is slightly moved manually without stopping the process from the coating to the non-coating zone of the aluminum rollers N°2 (Φ 20 mm) till the next sample coating in a series. 

**Figure 9:**
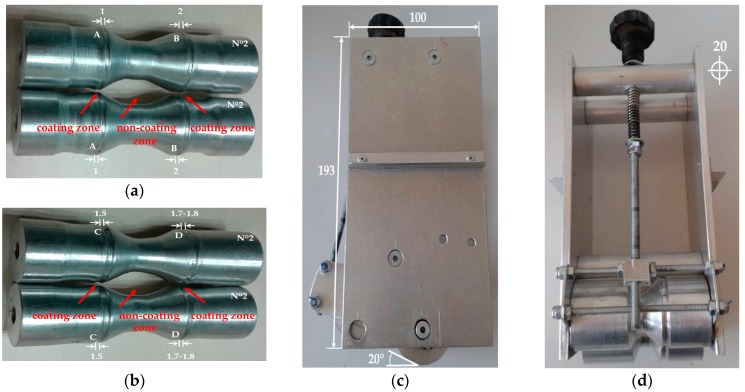
Roll to roll aluminum laboratory device parts: (**a**) rollers N°2 with notch widths A-A and B-B; (**b**) rollers N°2 with notch widths C-C and D-D; (**c**) lateral view and (**d**) front view.

Non-coating zone of the rollers N°2 was shaped in a way to obtain free movement of the yarn during the process after coating (Figure 9). For each coating step, rollers N°2 with notches width of 1 mm (A-first protective coating), 1.5 mm (C-first conductive coating), 1.7–1.8 mm (D-second conductive coating) and 2 mm (B-third conductive coating) has to be optimized. These rollers are not fixed onto the laboratory device and it is possible to change its notches’ position (pairs A-A, B-B, C-C or D-D). Two rollers “in pair” form a circular trajectory and give possibility for direct yarn going from a bath to the heating zone by obtaining equally coating distribution.

**Figure A2:**
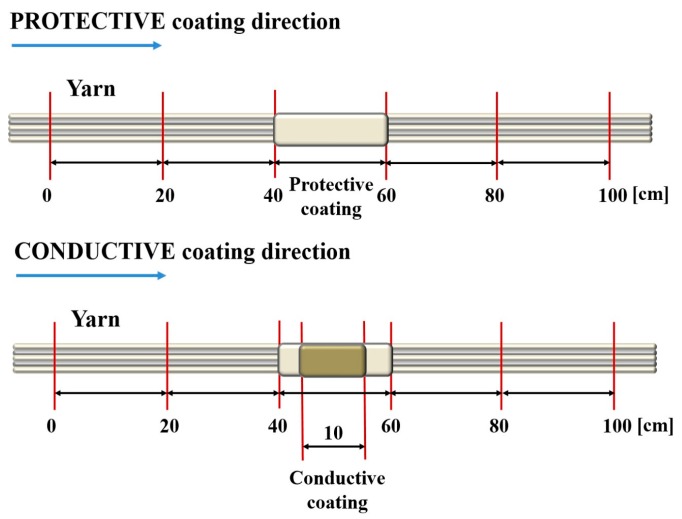
Coloured pen usage to mark given distance at sole yarn surface—scheme [53].

**Figure A3:**
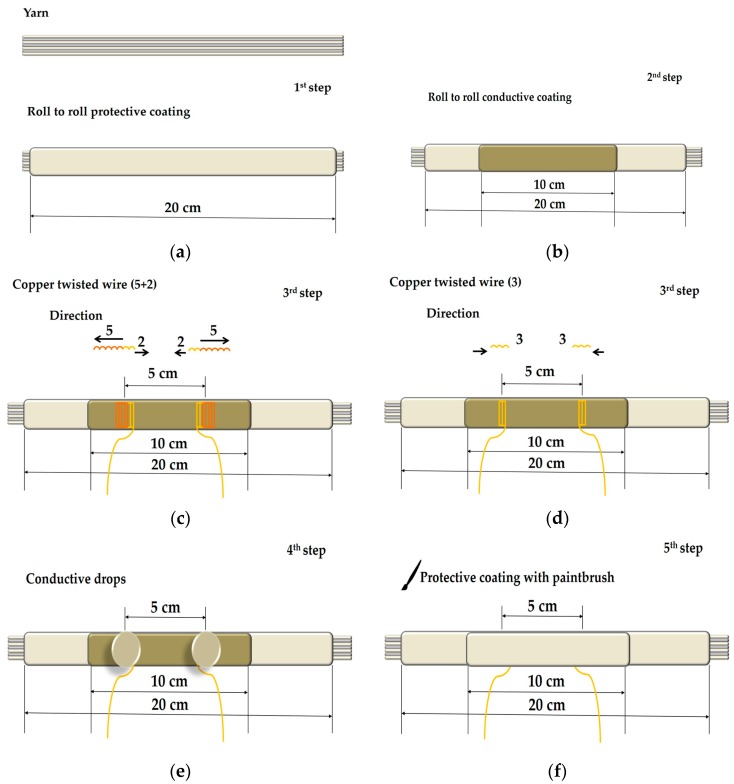
Production of one textile sensor—scheme: (**a**) roll to roll protective coating; (**b**) roll to roll conductive coating; (**c**) copper wire direction (5 + 2 rounds); (**d**) copper wire direction (3 rounds); (**e**) conductive drops addition; (**f**) last protective coating applied with paintbrush.

Hereafter, textile sensors were prepared by an original roll to roll coating method and a novel laboratory equipment under the defined protocol. The sensor preparation steps are presented in Appendix A (Figure A2 and Figure A3).

## 3. Results and Discussion

### Electromechanical Properties of Textile Sensors

In order to carry out electromechanical tests (electrical resistance variation during tensile testing of textile sensors developed (Table 6)), the tensile testing machine (MTS Systems Corporation, Eden Prairie, MN, USA) was used. Textile sensors were tested at the speed of 150 mm/min with a pre-load of 0.5 N. The distance between the clamps was 150 mm. The electrical resistance measurements were done by using a KUSB-3100 data acquisition digital I/O counter/timer (Keithley, Cleveland, OH, USA) and a simple resistance box connected to a computer (QuickDAQ software, Keithley, Cleveland, OH, USA). 

**Figure 10:**
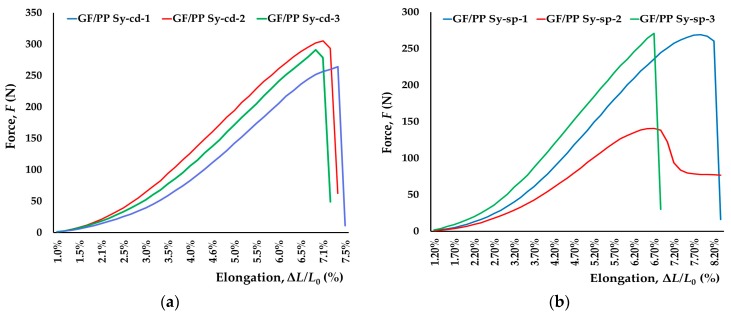

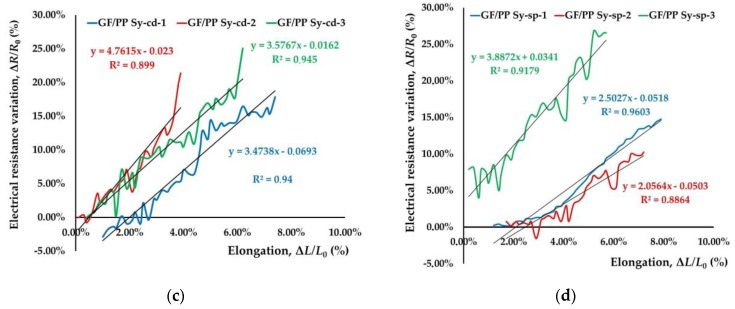
Electromechanical tests of GF/PP sensors developed by: (**a**,**c**) 8% PEDOT:PSS B/C drops; (**b**,**d**) silver drops added after copper wires insertion around conductive coated yarn.

Primarily, GF/PP sensors with 8% PEDOT:PSS B/C conductive drops, GF/PP Sy-cd, and with silver drops, GF/PP Sy-sp, added after copper wires insertion around conductive coating yarn were compared (Figure 10). Those conductive drops guarantee better electrical contact and smaller contact resistance.

**Figure 11:**
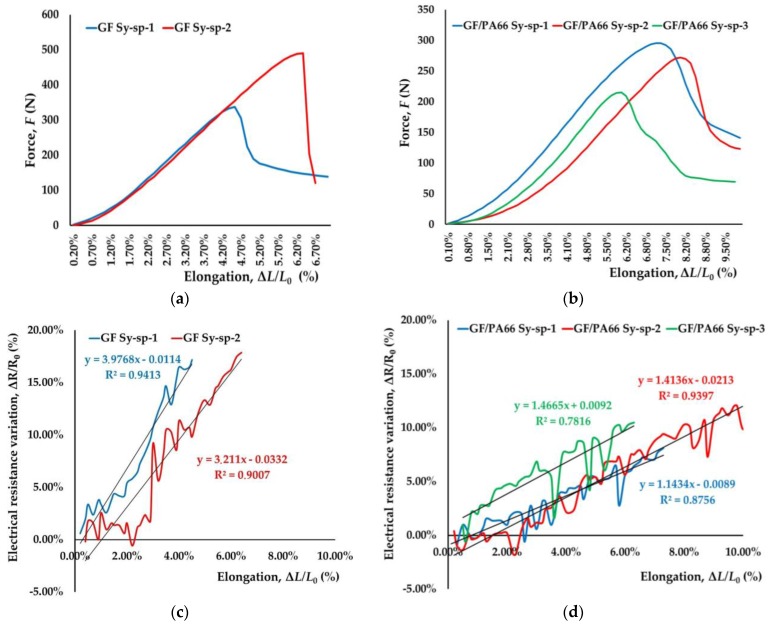
Electromechanical tests of textile sensors developed by silver drops added after copper wires insertion around conductive coated yarn: (**a**,**c**) GF Sy-sp sensors; (**b**,**d**) GF/PA66 Sy-sp sensors.

Other textile sensors were prepared with silver drops added during the preparation and electromechanically tested for in situ structural health monitoring of textile reinforced thermoplastic composites in real time (Figure 11).

**Figure 12:**
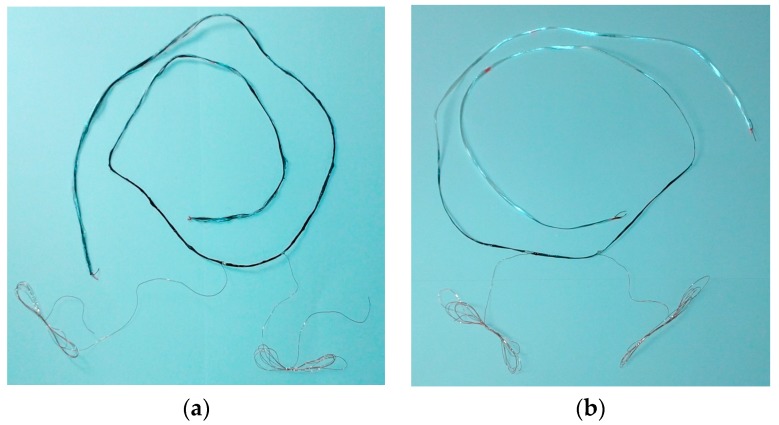
Textile sensors with silver drops added during preparation: (**a**) GF/PP sensor; (**b**) GF sensor.

Water resistance of these sensors is uncertain due to the several steps of their production and will be taken into greater consideration in our next study. Textile sensors show more uniform coating for produced GF sensors, GF Sy-sp, compared to GF/PP sensors, GF/PP Sy-sp and GF/PA66 sensors, GF/PA66 Sy-sp by visual perception after their production (Figure 12). This conclusion is supported by interface phenomena and related surface free energy (SFE) of textile sensors studied in a previous works [57,65]. According to Wu theory, SFE of GF/PP sensor is 47.55 mJ m^−2^ and SFE of GF/PA66 sensor is 35.65 mJ m^−2^. Lower SFE of GF sensor, 37.28 mJ m^−2^, than expected could be explained by a better coating process and “connection” (adhesion) of GF yarn and added coatings [65]. 8% PEDOT:PSS B/C conductive dry film prepared on the plexiglass surface shows also low SFE, 35.10 mJ m^−2^ [57].

When the textile sensor is stretched, two phenomena occur: the first one is related to the geometrical properties of the textile sensor; the cross-sectional area is decreasing, while the length is increasing; the sensor electrical resistance is increasing [1]. The second phenomena is related to the conductive layer made of PEDOT:PSS polymer complex and its electrical properties. As the concentration of the PEDOT:PSS is defined in order to be at the percolation threshold, the electrical conductivity is strongly decreasing when this layer is stretched, because a number of conductive paths inside the conductive material is broken. Therefore, this electrical conductivity is decreasing, or the electrical resistivity is increasing contributing to the increasing of the sensor electrical resistance together with “geometrical” increase of its resistance.

GF sensors, GF Sy-sp, show lower electrical resistance, ~850 Ω prior testing and lower elongation at break, 5.45%, higher force at break, 414.14 N, and higher gauge factor 3.5939 compared to GF/PP sensors, GF/PP Sy-sp (Table 7).

GF/PA66 sensors, GF/PA66 Sy-sp, show higher elongation at break, 7.20%, and lower gauge factor, 1.3412, compared to other textile sensors although number of copper twisted wires applied during their production has to be taken into account. Electrical resistance of GF/PA66 Sy-sp is slightly lower after production compared to other textile sensors, with lower dispersion of results.

Higher difference in electrical resistance values could not be observed at higher elongations, which confirms the coating uniformity achieved of treated yarns. Non-uniform coatings [66] tend to crack where there is a thin deposited layer. This causes a marked increase in electrical resistance whenever a conductive track breaks up.

## 4. Conclusions

The electrical resistivity of conductive dry films is an important parameter for the percolation threshold determination in order to define PEDOT:PSS content for textile sensor development. The electrical conductivity of PEDOT:PSS formulations after preparation started to decrease progressively during the 75 days observation period. Conductive dry films have granular morphology which confirmed that a more homogeneous surface resulted in higher electrical conductivity. According to the design concept study, an aluminum roll to roll laboratory device and a plexiglass chamber were realized and used for fibrous sensor production guaranteeing good quality and optimal coating repeatability. Textile sensors were prepared by an original roll to roll coating method and by a novel laboratory device following the defined protocol. A new generation of textile fibrous sensors based on PEDOT:PSS polymer complex are ready to be used for in situ structural health monitoring of textile reinforced thermoplastic composites in real time according to analysis of electromechanical measurements. GF sensors showed lower electrical resistance and elongation at break, higher force at break, and higher gauge factor compared to GF/PP sensors with silver drops added after copper wires insertion around conductive coated yarn. Those conductive drops guarantee good electrical contact and small contact resistance. GF/PA66 sensors indicated slightly lower electrical resistance after production, higher elongation at break, but lower gauge factor compared to other sensors.

## Figures and Tables

**Table 1:** Yarn and corresponding filament characteristics.

**Yarn**	**GF/PP**		**GF/PA66**		**GF**
Fineness (tex)	842.130		957.000		830.840
Diameter (mm)	0.798		0.821		0.638
Density (g/cm^3^)	1.682		1.797		2.600
Mass content (%)	71:29		65:35		100
Volume content (%)	46:54		45:55		100
**Filament**	**GF**	**PP**	**GF**	**PA66**	**GF**
Diameter (μm)	14.50	42.90	14.42	33.02	14.96
Number (%)	88	12	80	20	100
Density (g/cm^3^)	2.60	0.90	2.60	1.14	2.60

**Table 2:** List of chemicals for textile sensor production.

Nomenclature	Application	Chemical-Trade Name	Producer
A	Conductive coating	CLEVIOS P FORM. CPP105D	Heraeus, Leverkusen, Germany
B	Conductive coating	CLEVIOS F ET	Heraeus, Leverkusen, Germany
C	Protective coating	Latex Appretan 96100	Clariant, Paris, France
D	Wetting agent	NOVAROL DEL	Olea, Lodz, Poland

**Table 3:** List of other materials for textile sensors production.

Materials	Producer
Enamelled copper wire coil, Φ 0.20 mm	Conrad, Hirschau, Germany
Silver 5 g bottle paint conductive adhesive	RS Components, Corby, UK

**Table 4:** Technical data of chemicals used for yarn coating [55,56,57].

Chemical Technical Data	A	B	C
	Dispersion of PEDOT:PSS Polymer Complex, Organic Solvents and Polymeric Binders		Dispersion Based on Acrylic Esters Copolymer
	Mixture of Propan-2-ol (45%) and Water (55%)	Mixture of Ethanediol (10%) and Water (90%)	Self-Crosslinkable, Very Flexible, Hydrophobic, Free APEO
Solid content (%)	1.3	3.1	-
Appearance	liquid	liquid	liquid
Colour	blue	blue	milky-white
Density at 25 °C (g/cm^3^)	0.89	1	1.06
Concentration (%)	-	-	50
pH	3.0	Not determined	3.5
Brookfield viscosity at 20 °C and 100 s^−1^ (mPa·s)	30	55	100
Conductivity (Scm^−1^)	<300	~300	0
Dilutablity/Solubility	Fully miscible with water		

**Table 5:** Preparation of PEDOT:PSS aqueous dispersions.

**Mass of Aqueous Dispersion A or B**	**Mass of PEDOT:PSS in Aqueous Dispersion**	
	**A**	**B**
20 g	0.26 g	0.62 g
**PEDOT:PSS Content in Aqueous Dispersion A/B or B/C**	**Mass of Non-Conductive Aqueous Dispersion C**	
2%	12.74 g	30.38 g
10%	2.34 g	5.58 g
15%	1.47 g	3.51 g
20%	1.04 g	2.48 g
25%	0.78 g	1.86 g

**Table 6:** Textile sensor development.

Sample Label	Yarn	Number of Copper Twisted Wires	Conductive Drops
GF/PP Sy-cd	GF/PP	5 + 2	8% PEDOT:PSS B/C
GF/PP Sy-sp	GF/PP	5 + 2	Silver
GF/PA66 Sy-sp	GF/PA66	5 + 2	Silver
GF Sy-sp	GF	3	Silver

Additional description: Sy—sensor, cd—conductive drops added after copper wires insertion around conductive coated yarn, sp—silver drops added after copper wires insertion around conductive coated yarn.

**Table 7:** Electromechanical tests of textile sensors: sensor electrical resistance, tensile results and gauge factor.

Sample Label	Sensor Electrical Resistance		Force at Break (N)	Elongation at Break (%)	Gauge Factor
	After Production (Ω)	Prior Testing (Ω)			
GF/PP Sy-cd-1	930	2560	264.24	7.40	3.4738
GF/PP Sy-cd-2	950	2450	305.15	6.10	4.7615
GF/PP Sy-cd-3	920	2510	290.79	6.20	3.5767
Average	933	2507	286.73	6.57	3.9373
Standard deviation	15	55	20.76	0.72	0.7156
GF/PP Sy-sp-1	880	980	269.03	7.90	2.5027
GF/PP Sy-sp-2	700	800	140.80	7.20	2.0564
GF/PP Sy-sp-3	1540	1460	270.79	5.70	3.8872
Average	1040	1080	226.87	6.93	2.8154
Standard deviation	442	341	74.55	1.12	0.9546
GF Sy-sp-1	1240	1200	337.69	4.50	3.9768
GF Sy-sp-2	500	500	490.60	6.40	3.2110
Average	870	850	414.14	5.45	3.5939
Standard deviation	523	495	108.13	1.34	0.5415
GF/PA66 Sy-sp-1	990	1150	295.41	7.30	1.1434
GF/PA66 Sy-sp-2	810	930	271.85	8.00	1.4136
GF/PA66 Sy-sp-3	690	820	214.93	6.30	1.4665
Average	830	967	260.73	7.20	1.3412
Standard deviation	151	168	41.38	0.85	0.1733

## Appendix A

### Appendix A.1. Roll to Roll Coating Method

The yarn movement is controlled with two motor devices placed at the ends of the coating path. The duration of yarn introduction in a bath (135 mL of dispersion) at each coating step has been determined precisely in order to avoid depositing used dispersions on aluminum rollers (Figure A1) [52]. A slower coating speed, 0.2 m/min, must be used during the process due to later slower drying of the coated yarn. A temperature of 170 °C is used for the heating system, HG 2310 LCD programmable intellitemp™ heatgun (Steinel Professionel, Herzebrock-Clarholz, Germany), at a distance of less than 5 cm from the coated yarn. The left N°1 rollers (Φ 40 mm) after pre-testing were finally set up at an angle of 20° between the aqueous dispersion surface in a plexiglass chamber and the right N°1 rollers to prevent early yarn coating.

### Appendix A.2. Textile Sensors Preparation

A coloured pen is used to mark the given distance at sole yarn surface (Figure A2). It is possible to make ten or more textile sensors in one series, but final product quality must be taken into account. After each 1 m of yarn in a series short length (30 mm) of PP yarn is twisted around the yarn to recognize each new sample during the coating. Besides, additional yarn length must be taken for (sensor) yarn placement (raw bobbin and coated bobbin) on the two motor devices, M1 and M2, approximately 3 m on each side.

The textile sensors in a series were prepared according to description below:

(i) The sole yarn was coated with protective coating, chemical C, (Figure A3a). One drop of fast-acting anionic wetting agent, NOVAROL DEL, chemical D, used for the treatment of cotton, wool and synthetic fibres was added in C-aqueous dispersion for the first protective coating to improve homogeneity of yarn coating and to increase the adhesion between the conductive coating and yarn as a following step for textile sensors production.
(ii) After that, two conductive coatings, 8% PEDOT:PSS B/C dispersion, were applied according to the preliminary study as the optimum number of conductive coatings (Figure A3b).
(iii) Copper wires were twisted around conductive coated yarn, 5 rounds in one direction and 2 in the opposite direction, to avoid electrical disturbance due to inductivity and capacity of the connections (Figure A3c), or 3 rounds in one direction (Figure A3d), before the last protective coating and placed at the distance of 5 cm.
(iv) Conductive drops of 8% PEDOT:PSS B/C mixture or silver were added after wires insertion (Figure A3e) around conductive coated yarn due to connection improvement between them.
(v) The last protective coating was applied on the treated yarn(s) with paintbrush (Figure A3f).
(vi) Samples were dried overnight under standard conditions (20 °C, 65% R.H.).
